# Combining Immunoassays to Identify Zika Virus Infection in Dengue-Endemic Areas

**DOI:** 10.3390/tropicalmed7100254

**Published:** 2022-09-21

**Authors:** Pichamon Sittikul, Pimolpachr Sriburin, Jittraporn Rattanamahaphoom, Kriengsak Limkittikul, Chukiat Sirivichayakul, Supawat Chatchen

**Affiliations:** Department of Tropical Pediatrics, Faculty of Tropical Medicine, Mahidol University, Bangkok 10400, Thailand

**Keywords:** Zika virus, dengue virus, nonstructural protein 1, serological diagnosis, cross-reactivity

## Abstract

Zika virus (ZIKV) is a mosquito-borne flavivirus that has recently emerged as a global health threat. The rise in ZIKV infections has driven an increased incidence of neonates born with microcephaly or other neurological malformations. Therefore, screening for ZIKV infection can considerably impact pregnant women, especially during the first trimester. The majority of ZIKV infections are mild or asymptomatic, and clinical diagnosis is inaccurate. Moreover, given the high level of cross-reactivity among flaviviruses, serological approaches to distinguish ZIKV from dengue virus (DENV) infections are complicated. We used the combination of DENV and ZIKV nonstructural protein 1 (NS1) IgG enzyme-linked immunosorbent assay (ELISA) and ZIKV NS1 blockade-of-binding (BOB) ELISA to test the convalescent sera of non-flavivirus, primary DENV, secondary DENV, and ZIKV infections. Our findings indicate that primary testing using a ZIKV NS1 IgG ELISA, the test of choice for large-scale ZIKV serosurvey studies, provided relatively high sensitivity. Moreover, the confirmation of positive ELISA results using the ZIKV NS1 BOB ELISA increased average specificity to 94.59% across serum samples. The combined use of two simple ELISAs for ZIKV serosurveys and the monitoring of ZIKV infection during pregnancy can elucidate the epidemiology, pathogenesis, and complications of ZIKV in DENV-endemic areas.

## 1. Introduction

Zika virus (ZIKV) is an enveloped RNA virus in the family *Flaviviridae*, genus *Flavivirus* and is similar to other clinically relevant flaviviruses (e.g., dengue virus (DENV), Japanese encephalitis virus, and yellow fever virus) [1]. ZIKV is transmitted by mosquitoes, primarily the *Aedes aegypti* and the *Aedes albopictus*. ZIKV can be further transmitted through sexual, mother-to-fetus, and blood transfusion routes [2]. With a genome length of approximately 11 kb, the ZIKV is a positive-sense, single-stranded RNA virus that codes for three structural proteins—capsid, pre-membrane, and envelope—and seven nonstructural proteins: nonstructural protein 1 (NS1), NS2a, NS2b, NS3, NS4a, NS4b, and NS5b [3]. NS1 is involved in viral replication, immune evasion, and pathogenesis and is considered an important antigenic marker of ZIKV and other flaviviruses [4]. Antibodies to NS1 have high sensitivity and limited cross-reactivity, suggesting that NS1 may represent an efficient differential assay for DENV and ZIKV infections [5]. ZIKV was originally identified in 1947 in a rhesus monkey in the Zika Forest of Uganda. From there, ZIKV has spread slowly throughout Africa and Asia. The first outbreak of ZIKV infection occurred in 2007 in Yap State, Micronesia, in the western Pacific Ocean [6,7]. The first report on the possible presence of ZIKV in Thailand was published in 1963 [8]. In 2016, small outbreaks of ZIKV infection were reported in Singapore [9], and several hundred cases were detected in Thailand in the same year [10].

ZIKV infections generally cause mild and self-limited illness. However, the infection can cause Guillain–Barré syndrome in adults and microcephaly in infants born to ZIKV-infected women [11]. Therefore, screening for ZIKV infections can considerably impact pregnant women, especially during the first trimester [10,12]. The diagnosis of acute ZIKV infection is made through the detection of its viral components (RNA or viral NS1 proteins) within 2 weeks of illness [13]. ZIKV RNA can be detected in a variety of specimens, including blood, serum, plasma, saliva, semen, and cerebrospinal fluid. The detection of viral components is highly sensitive and specific, but the effectiveness of the ZIKV RNA assay is limited by the short period of viremia [14]. Further, serological assays for ZIKV antibodies are widely used for diagnosis, particularly after 2 weeks of the onset of illness, in seroprevalence studies, or in prenatal screening. Unfortunately, ZIKV’s cross-reactivity with other antigenically similar flaviviruses—DENV in particular—is a challenging problem in areas such as Thailand, where these viruses co-circulate and where ZIKV infection is considerably less prevalent than DENV infection [15]. Therefore, positive serological test results should be confirmed with tests—such as the plaque reduction neutralization test (PRNT)—that use an alternative platform. However, the PRNT is low-throughput and requires a longer turnaround time (3–7 days) compared with ELISA. Moreover, the need for experienced and highly trained personnel makes the use of the PRNT challenging in a high-volume clinical test setting [1,13,16]. Considerable research has been conducted to develop serological strategies—including the development of the ZIKV NS1 IgG enzyme-linked immunosorbent assay (ELISA) [17,18], the blockade-of-binding (BOB) ELISA [16,19], the microneutralization assay [16], and a cytopathic effect-based virus neutralization test—for more specific ZIKV antibody detection in DENV-endemic areas [15]. In this study, we identified an efficient and practicable strategy that can be used in large-scale ZIKV seroprevalence studies. The strategy relies on primary testing using NS1 IgG ELISA followed by a ZIKV NS1 BOB ELISA for confirmation of positive ELISA results. 

## 2. Materials and Methods

### 2.1. Serum Samples

The convalescent sample (1–2 weeks after symptoms onset) in this study was selected from a cohort study of the epidemiology of dengue disease conducted from 2006 to 2009 in school-aged children in Ratchaburi province, Thailand [20]. We used 10 human convalescent sera with primary DENV infections (50% male, age less than 15 years old, DENV serotype 1–4) and 21 human convalescent sera with secondary DENV infections (61.9% male, age less than 15 years old, DENV serotype 1–4) that had a detectable DENV infection by reverse transcription PCR (RT-PCR). Six human convalescent sera with non-flavivirus infection (tested by ELISA and PRNT) were included in the study. 

All 30 ZIKV-positive samples (1–2 weeks after symptoms onset) were selected from a study of Zika incidence in dengue epidemiology study in Ratchaburi province, Thailand [20,21]. All samples showed seropositivity using ZIKV NS1 IgG ELISA, and ZIKV infection was confirmed by RT-PCR in acute sera [21]. All procedures were ethically approved before the study (TMEC 21-013). 

### 2.2. Production of ZIKV and DENV NS1

Full-length NS1 genes from ZIKV strain SV0127 and DENV serotypes 1, 2, 3, and 4 with NCBI accession numbers KU681081.3, AF180817.1, KU725663.1, KU725665.1, and M14931.2, respectively, were amplified using SuperScrip III One-Step RT-PCR (Invitrogen, Thermo Fisher Scientific, Inc., Waltham, MA, USA) and cloned into pET vectors (Novagen, Sigma-Aldrich, Burlington, MA, USA). The NS1 proteins were over-expressed in *E. coli* BL21 (DE3) in Luria Broth (LB) medium supplemented with 50 µg/mL kanamycin. The optimal conditions for protein expression included keeping cells at 25 °C overnight and inducing them with 1 mM isopropyl β-D-1-thiogalactopyranoside (IPTG) (Bio Basic, Amherst, NY, USA). After expression, cells were harvested by centrifugation at 4000 rpm for 30 min at 4 °C. Cell pellets were resuspended in 1×PBS and were subsequently sonicated by Sonics Vibra Cell VCX750 (Sonics & Material, Inc., Newtown, CT, USA) for 30 min (pulse on 5 s; pulse off 5 s). After sonication, the cell lysate was centrifuged at 10,000 rpm for 30 min at 4 °C. The insoluble protein was solubilized in denatured buffer (50 mM Tris-HCl pH 8, 0.5 M NaCl, 0.03 M imidazole, and 8 M urea). Proteins were purified using the HisTrap column (GE Healthcare, Chicago, IL, USA) under denaturing conditions. The purities of NS1 proteins with a molecular weight of 40 kDa were analyzed using 12% SDS-PAGE. Protein concentrations were measured using a NanoDrop spectrophotometer (Thermo Fisher Scientific, Inc., Waltham, MA, USA), and proteins were kept at −80 °C until used. 

### 2.3. Indirect ELISA

Indirect ELISA was performed using a previously described standard protocol [21,22]. In brief, plates were sensitized with ZIKV NS1 (10 µg/mL or 500 ng/well), DENV 1–4 NS1 (5 µg/mL, 250 ng for each serotype or a total of 1000 ng/well), or inactivated ZIKV (MR766) in PBS buffer and incubated at 37 °C overnight. After incubation, plates were washed six times with PBS containing 0.05% Tween20 (200 µL/well) and blocked at 4 °C overnight with 200 µL of PBS containing 5% skim milk. After blocking, plates were washed six times with PBS containing 0.05% Tween20 (200 µL/well), 50 µL of tested serum (dilution 1:1000) was added, and plates were incubated at 37 °C for 1 h. Then, plates were washed six times with PBS containing 0.05% Tween20 (200 µL/well), 50 µL conjugate (goat anti-human IgG-HRP, dilution 1:10,000) was added, and plates were incubated at 37 °C for 1 h. After incubation, plates were washed six times with PBS containing 0.05% Tween20 (200 µL/well), 100 µL substrate (SureBlue TMB microwell peroxidase) was added, and plates were incubated for 30 min at room temperature. The reaction was stopped by adding 0.4 M H_2_SO_4,_ and the absorbance was read at 450 nm. P/N ratio is the ratio of the average OD_450_ of the test sample divided by the average OD_450_ of the negative sample. The P/N ratio ≥ 2 was considered to be positive. 

### 2.4. ZIKV NS1 BOB ELISA

ZIKV NS1 BOB ELISA was performed as described in a previous report [19]. The ELISA microplate (Greiner Bio-One, Kremsmünster, Austria) was coated overnight at 4 °C with 1 µg/mL ZIKV NS1 MR766 strain (Aviva Systems Biology, San Diego, CA, USA; 50 µL/well). After incubation, plates were washed twice with PBS containing 0.05% Tween20 (200 µL/well) and blocked at room temperature for 1 h with PBS containing 1% BSA (200 µL/well). After blocking, plates were washed twice with PBS containing 0.05% Tween20 (200 µL/well). Then, 50 µL serum (1:10 dilution) or positive control ZKA35 (Absolute Antibody, UK) was added to ZIKV NS1-coated ELISA plates, and plates were incubated for 1 h at room temperature. Then, 50 µL of ZKA35-HRP conjugate (Absolute Antibody, UK) was added, and the mixture was incubated at room temperature for 1 h. After incubation, plates were washed four times with PBS containing 0.05% Tween20 (200 µL/well). After washing, 50 µL substrate (SureBlue TMB microwell peroxidase substrate) was added, and plates were incubated for 30 min at room temperature. The reaction was stopped by adding 0.4 M H_2_SO_4,_ and the absorbance was read at 450 nm. The % inhibition was calculated according to the following equation:

% inhibition = ([OD sample − OD neg ctr]/[OD pos ctr − OD neg ctr]) × 100

## 3. Results

### 3.1. Analysis of Convalescent Samples of DENV-Infected Patients

**Figure 1:**
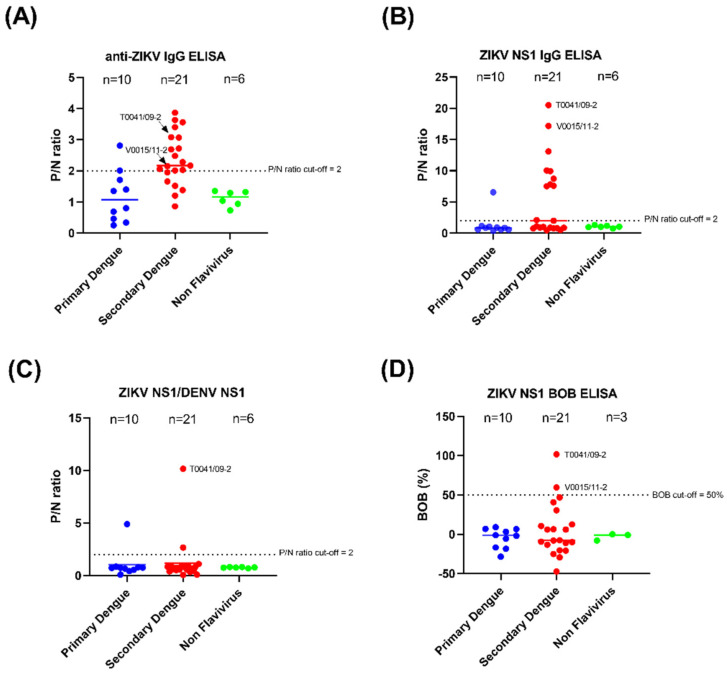
The performance of serological assays using DENV infection samples in the convalescent phase. (**A**) anti-ZIKV IgG ELISA. (**B**) ZIKV NS1 IgG ELISA. (**C**) ZIKV NS1/DENV NS1 IgG ELISA. (**D**) ZIKV NS1 BOB ELISA. The dotted line represents the cut-off value of the P/N ratio or %BOB inhibition. ZIKV: Zika virus, DENV: dengue virus, ELISA: enzyme-linked immunosorbent assay, BOB: blockade-of-binding, NS1: nonstructural protein 1, P/N ratio: ratio of average OD_450_ of test sample divided by the average OD_450_ of the negative sample.

In this study, specificity and cross-reactivity were further evaluated using convalescent serum samples derived from DENV-infected patients. The ELISA using the whole ZIKV antigen poorly identified DENV infections, particularly in secondary DENV infection (Figure 1A, Table 1). In the ZIKV NS1 IgG ELISA, one serum sample (10%) from the primary DENV infection group showed cross-reactivity, whereas 10 out of 21 samples (47.6%) from the secondary DENV infection group cross-reacted with ZIKV NS1 (Figure 1B, Table 1). In combined use of the ZIKV and DENV NS1 IgG ELISA, furthermore, the cross-reaction was decreased when the P/N ratio of ZIKV NS1/DENV-NS1 was used. Only two samples (9.52%) of secondary DENV infection showed cross-reactivity with ZIKV (Figure 1C). 

The BOB ELISA relies on the ability of serum antibodies to block the binding of a monoclonal antibody (mAb) to an antigen adsorbed on a microtiter ELISA plate; this assay showed high specificity [19]. Interestingly, the specificity was 90.48% when secondary DENV samples were tested with the ZIKV NS1 BOB ELISA (Figure 1D; Table 2). The combined use of the P/N ratio of ZIKV-NS1/DENV NS1 and the ZIKV NS1 BOB test distinguished ZIKV infections from DENV infections.

### 3.2. Analysis of Zika Infection Samples in the Convalescent Phase

**Figure 2:**
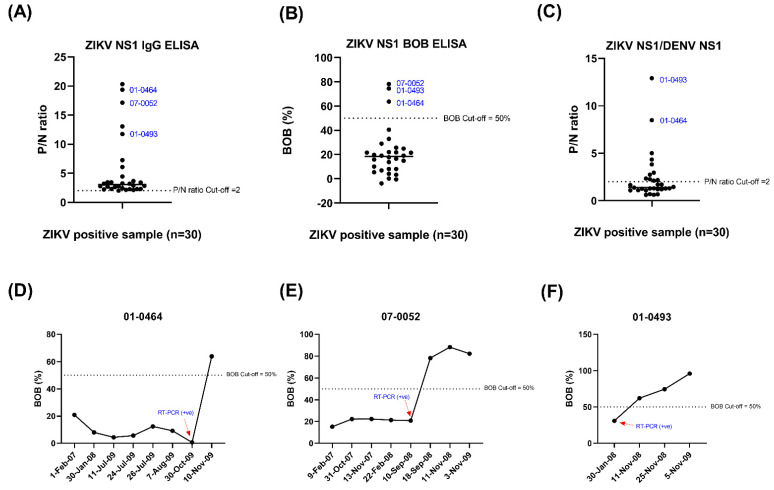
Performance of serological assay using ZIKV infection samples in the convalescent phase. (**A**) ZIKV NS1 ELISA. The dotted line represents the cut-off value of the P/N ratio. (**B**) ZIKV NS1 BOB ELISA. The dotted line represents the cut-off value of % BOB inhibition. (**C**) ZIKV NS1/DENV NS1 ELISA. The dotted line represents the cut-off value of the P/N ratio. (**D**–**F**) ZIKV NS1 BOB ELISA in a sequential annual blood sample of three ZIKV infection patients (01-0464, 07-0052, and 01-0493). The red arrow indicates the date of ZIKV RT-PCR positivity. The dotted lines represent the cut-off value of % BOB inhibition. ZIKV: Zika virus, DENV: dengue virus, ELISA: enzyme-linked immunosorbent assay, BOB: blockade-of-binding, NS1: nonstructural protein 1, P/N ratio: ratio of average OD_450_ of test sample divided by the average OD_450_ of the negative sample.

Recently, our group reported using ZIKV NS1 IgG ELISA screening to identify symptomatic ZIKV infections [21]. However, only the sample with a high P/N ratio (>10) showed a positive score with the ZIKV NS1 BOB ELISA, as shown in Figure 2A,B. We performed a longitudinal study to gain more knowledge of Zika antibody dynamics and the duration of protection after ZIKV infection. We tested three annual blood samples (01-0464, 07-0052, and 01-0493) that were positive for the ZIKV NS1 BOB assay and observed that the antibody can be detected after one year (Figure 2C,D).

Our study showed that the ZIKV NS1 BOB test was highly specific in discriminating ZIKV infection from DENV infection and was recommended as the test for confirmation; however, the sensitivity was low compared to that of the ZIKV NS1 IgG ELISA. The combined use of the ZIKV NS1 IgG and the DENV NS1 IgG ELISAs can provide an alternative approach to better determine ZIKV serostatus in DENV-endemic areas. 

## 4. Discussion

The serological diagnosis of ZIKV is challenging in dengue-endemic areas. Our study concluded that an effective testing method remains unavailable. The ZIKV IgG and ZIKV NS1 IgG ELISAs provide high sensitivity but low specificity (54% for ZIKV IgG and 70% for ZIKV NS1 IgG), particularly in patients with secondary DENV infection. The low specificity can be explained by the cross-reactivity between DENV and ZIKV antibodies [4]. The lower specificity in patients after secondary DENV infection can be attributed to the characteristically broader antibody response to the antigens of the flavivirus group after secondary DENV infection, while the antibody response after primary DENV infection is predominantly against the type-specific determinant [23]. The sensitivity of the ZIKV NS1 BOB test was lower than that of the ZIKV NS1 IgG ELISA, consistent with a previous study indicating that the sensitivity of a ZIKV NS1 BOB ELISA using F9 mAb was lower than that of the Zika microneutralization assay, especially in samples collected more than 6 months post-infection [16]. The high specificity (90%) of the ZIKV NS1 BOB ELISA is the test’s strength. Our results indicated that the ZIKV NS1 BOB ELISA showed no cross-reactivity with primary DENV samples and non-flavivirus infection and that the antibody can be detected after more than one year. However, the ZIKV NS1 BOB ELISA requires monoclonal antibodies and commercial NS1 proteins [19]. In addition, the combined use of ZIKV and DENV NS1 ELISAs was applied to distinguish ZIKV from previous DENV infections [17]. A test using the P/N ratio of ZIKV NS1/DENV NS1 provided specificity comparable to that of the ZIKV BOB ELISA and higher sensitivity (30%); however, this level of sensitivity remains unacceptably low. Primary testing using an NS1 IgG ELISA followed by a ZIKV NS1 BOB ELISA for confirmation of positive ELISA results can decrease the cost of expensive ZIKV NS1 BOB ELISA testing but does not increase sensitivity. Given that the ZIKV NS1 IgG ELISA is cost-effective because it uses an in-house NS1 protein and provides relatively high sensitivity and specificity, we propose this ELISA as the test of choice for Zika seroepidemiological studies. In areas where the incidence of ZIKV is very low, and that of DENV is very high, confirmation of ZIKV infection using the ZIKV NS1 BOB test or the P/N ratio of ZIKV NS1/DENV NS1 can be applied to increase the study accuracy.

The limitations of this study were also considered. The study samples were taken from the previously archived serum from the children in the dengue-endemic area, and the sample numbers for each group were limited.

## 5. Conclusions

This study supports that the combined use of two simple ELISAs (DENV NS1 and ZIKV NS1) for ZIKV serosurveys and the monitoring of ZIKV infection during pregnancy can elucidate the epidemiology, pathogenesis, and complications of ZIKV in DENV-endemic areas. 

## Figures and Tables

**Table 1:** Results of enzyme-linked immunosorbent assays in various serum panels.

Sample/Test	ZIKV IgG			ZIKV NS1-IgG			ZIKV NS1-IgG /DENV NS1-IgG			BOB			ZIKV NS1-IgG followed by BOB		
	+	−	%	+	−	%	+	−	%	+	−	%	+	−	%
**Non-flavivirus** **(*n* = 6)**	0	6	0%	0	6	0%	0	6	0%	0	3	0%	0	3	0%
**Primary DENV** **(*n* = 10)**	2	8	20%	1	9	10%	1	9	10%	0	10	0%	0	10	0%
**Secondary DENV (*n* = 21)**	15	6	71.4%	10	11	47.6%	2	19	9.52%	2	19	9.52%	2	19	9.52%
**ZIKV** **(*n* = 30)**	30	0	100%	30	0	100%	9	21	30%	3	27	10%	3	27	10%

**Table 2:** Sensitivity and specificity to ZIKV of enzyme-linked immunosorbent assays.

ELISA	Sensitivity (95% CI)	Specificity		
		In Primary DENV Samples (95% CI)	In Secondary DENV Samples (95% CI)	In Overall Serum Samples (95% CI)
**ZIKV IgG**	100% (88.7–100%)	80% (49.0–96.5%)	28.57% (13.8–50%)	54.05% (38.4–69%)
**ZIKV NS1-IgG**	100% (88.7–100%)	90% (59.6–99.5%)	52.4% (32.4–71.7%)	70.27% (54.2–82.5%)
**ZIKV NS1-IgG/DENV NS1-IgG**	30% (16.7–47.9%)	90% (59.6–99.5%)	90.48% (71.1–98.3%)	91.89% (78.7–97.2%)
**BOB**	10% (3.5–25.6%)	100% (72.3–100%)	90.48% (71.1–98.3%)	94.59% (82.3–99%)
**ZIKV NS1-IgG followed by BOB**	10% (3.5–25.6%)	100% (72.3–100%)	90.48% (71.1–98.3%)	94.59% (82.3–99%)

## Data Availability

Not applicable.
